# The evolving role of endoscopy in the diagnosis of premalignant gastric lesions

**DOI:** 10.12688/f1000research.12087.1

**Published:** 2018-06-08

**Authors:** William Waddingham, David Graham, Matthew Banks, Marnix Jansen

**Affiliations:** 1 Department of Endoscopy, University College London Hospital, London, UK; 2 UCL Cancer Institute, University College London, London, UK; 3 Department of Pathology, University College London, London, UK

**Keywords:** gastric cancer, endoscopy, premalignant lesions

## Abstract

Gastric adenocarcinoma is a disease that is often detected late, at a stage when curative treatment is unachievable. This must be addressed through changes in our approach to the identification of patients at increased risk by improving the detection and risk assessment of premalignant changes in the stomach, including chronic atrophic gastritis and intestinal metaplasia. Current guidelines recommend utilising random biopsies in a pathology-led approach in order to stage the extent and severity of gastritis and intestinal metaplasia. This random method is poorly reproducible and prone to sampling error and fails to acknowledge recent advances in our understanding of the progression to gastric cancer as a non-linear, branching evolutionary model. Data suggest that recent advances in endoscopic imaging modalities, such as narrow band imaging, can achieve a high degree of accuracy in the stomach for the diagnosis of these premalignant changes. In this review, we outline recent data to support a paradigm shift towards an endoscopy-led approach to diagnosis and staging of premalignant changes in the stomach. High-quality endoscopic interrogation of the chronically inflamed stomach mucosa, supported by targeted biopsies, will lead to more accurate risk assessment, with reduced rates of under or missed diagnoses.

## Introduction

Gastric adenocarcinoma remains a major source of cancer-related mortality. Despite recent declines in incidence, it is the fifth most common cancer worldwide ^1, 2^ and the fourth most common cause of cancer-related death in Europe ^1^. It is of considerable concern that prognosis remains dismal, with reported UK 5-year survival rates of 20% in men and 18% in women as recently as 2011 ^3^. Around a third (32%) of gastric cancer cases in England are diagnosed through an emergency route of presentation ^4^. A considerable proportion (46–57%) of patients have advanced disease (stage 4) at the time of diagnosis ^3^. Of note, recent studies have demonstrated an increasing incidence of gastric adenocarcinoma among young white people in the US, with a Swedish study demonstrating an increasing trend of atrophic gastritis among adults aged 35–44. These data suggest that the well-documented decline in rates of gastric cancer may change ^5, 6^. Japan’s earlier stage of diagnosis and superior 5-year survival highlight the need for better pathways to early diagnosis and treatment in order to improve on the current poor prognosis ^7^.

A recent retrospective analysis by Chadwick *et al.* suggested that 8.3% of gastric cancers were missed at endoscopy ^8^. In a similar study, Menon *et al.* demonstrated that 11.3 % of upper gastrointestinal (GI) cancers are missed at endoscopy, up to 3 years before diagnosis ^9^. A meta-analysis estimated the rate of missed gastric cancer to be 9.4% from a cohort of 22 studies ^10^. These studies suggest that the quality of diagnostic upper GI endoscopy in this context should be a target for improvement. Currently, the diagnosis and risk stratification of premalignant changes in the stomach, such as chronic atrophic gastritis (CAG) and gastric intestinal metaplasia (GIM), are reliant on histopathology. However, there has been considerable progress in advanced endoscopic imaging techniques and their use in identifying these premalignant changes. An understanding of the pathogenesis of gastric cancer, and the risk associated with pre-malignant lesions, including CAG and GIM, is vital to ensuring high-quality endoscopic diagnostic care. In this review, we outline the evidence for a change in paradigm, towards an endoscopy-led model, for diagnosing and staging pre-malignant lesions in the stomach. This approach in tandem with directed, non-random histopathological sampling could improve secondary prevention through more accurate, evidence-based screening and surveillance.

## Pathogenesis of cancer in the chronically inflamed stomach, a branching evolutionary model

**Figure 1.**
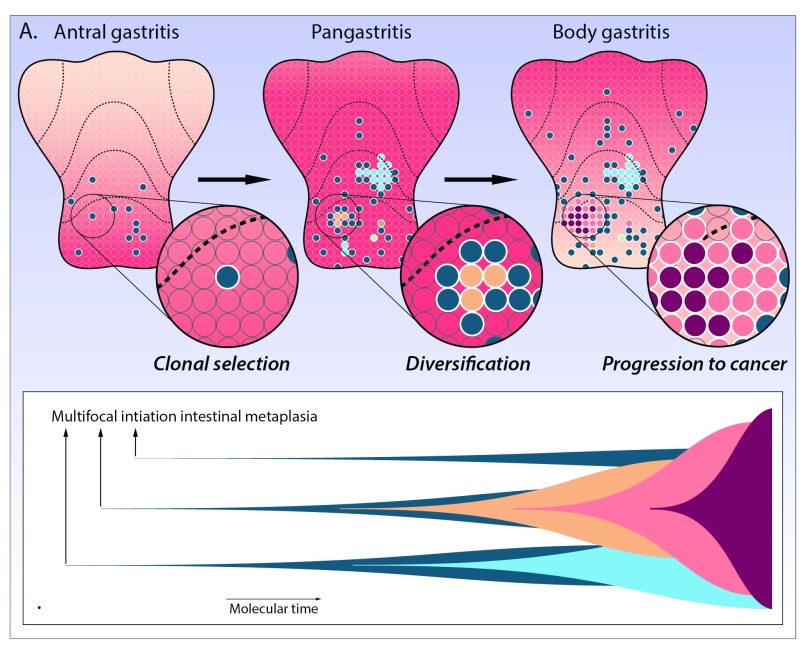
Evolution to cancer in the chronically inflamed stomach. **A**) In this diagram, every gastric stem cell niche is represented by an individual circle. Gastric mucosal inflammation is indicated by the red shading, and each metaplastic clone is demonstrated by a coloured circle. The scenario outlined in the main text is played out across the chronically inflamed stomach, driving the erratic emergence of countless numbers of clones and subclones and dividing the stomach mucosa into a mosaic of competing clones battling for space. Clones expand through gland duplication. This clonal diversification and competition scenario is driven by increasing mucosal inflammation spreading like a wave-front along the gastric mucosa (indicated by the red shading) from distal to proximal with advancing disease stages. This underpins continued selection of metaplastic clonal lineages, as shown in the consecutive panels. All coloured circles show patches of intestinal metaplasia that expand and genetically diverge with increasing disease stages (left to right). **B**) Muller plot showing this branching clonal evolution scenario in the chronically inflamed stomach. With time, multiple independent metaplastic clones are initiated, which expand and compete for space in the gastric mucosa. During clonal expansion, further random mutations are inevitably acquired, some of which may drive the selection and expansion of subclones. Rare clones may progress to gastric adenocarcinoma (colours correspond to lineages shown in **A**).

Pelayo Correa and colleagues described a linear cascade for gastric cancer pathogenesis, emphasising the gradual transformation of gastric-type epithelium to intestinal-type epithelium and finally invasive cancer, in a progression driven by dietary nitroso compounds ^11^. Later descriptions advanced our understanding by embracing the role of *Helicobacter pylori* as the primary causative environmental agent ^12, 13^. Furthermore, Epstein-Barr virus (EBV) may play a role in a pathogenesis that is molecularly distinct in a small subset (9%) of gastric adenocarcinoma cases ^14, 15^. Here, we adapt the classical linear Correa progression sequence by incorporating a modern understanding of clonal evolution in glandular epithelial tissues and outline a branching evolutionary ‘adaptation and selection’ model ( Figure 1). Please note that this review will focus mostly on gastric cancer pathogenesis in the chronically inflamed stomach. Other settings wherein gastric cancer may evolve, such as on a familial background provoking diffuse-type gastric cancer, will not be discussed here. We refer the interested reader to other excellent reviews on this topic ^16, 17^.

The sequence of progression of gastric adenocarcinoma in the context of CAG is fundamentally different to that described in the development of adenocarcinoma of the colon. In the stomach, the entire mucosal lining is effectively transformed into a pre-neoplastic field, and demarcation of early pre-malignant lesions is not nearly so distinct as compared to the polyp-carcinoma sequence in the colon. The two main histopathologic precursor lesions that precede the development of neoplasia in gastric cancer progression are intestinal metaplasia and gastric atrophy. These will be discussed in turn.

**Figure 2.**
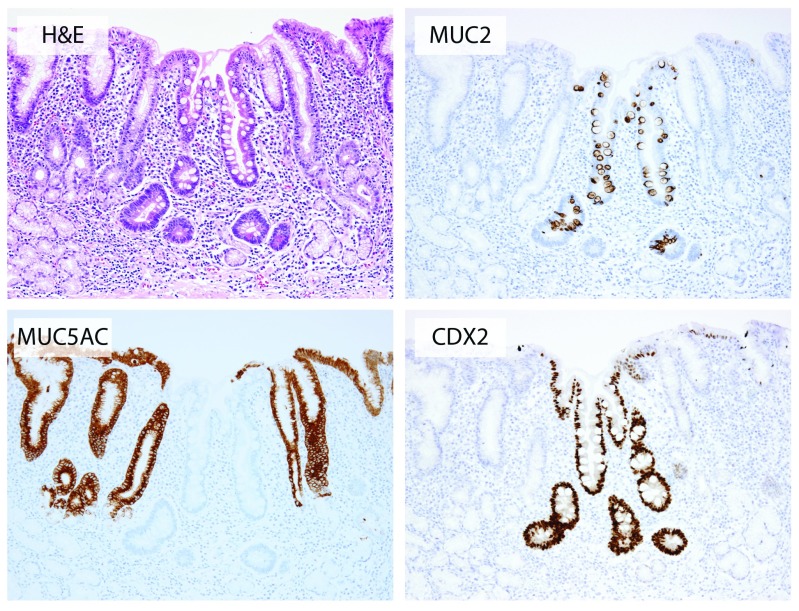
Histopathology of gastric intestinal metaplasia. A microscopic focus of intestinal metaplasia in chronically inflamed gastric mucosa with moderate glandular atrophy in the background. Note the abrupt transition from intestinal metaplastic epithelium to pre-existent foveolar epithelium. The gland arrangement is distorted and there is a complete loss of differentiated cell types such as parietal and chief cells, consistent with functional atrophy. The other panels show MUC2 and CDX2 marking intestinal lineages and MUC5AC marking gastric foveolar lineages (clockwise).

Evolution to cancer is invariably a process of somatic clonal evolution of stem cells through the natural selection of randomly generated, advantageous phenotypic traits ^18^. McDonald *et al.*’s landmark study published in 2008 significantly furthered our understanding of the progression to gastric adenocarcinoma by demonstrating that intestinal metaplasia is a clonally derived alteration within the gastric mucosa ^19^. These authors showed that each gland within a given patch of GIM carried the same somatic marker mutation, whilst surrounding (pre-existent) gastric glands did not carry this somatic marker mutation. This lineage-tracing experiment established indisputable evidence that the development of intestinal metaplasia is a change that occurs at the level of the tissue-specific stem cell. The fact that somatic genetic alterations are shared across patches of intestinal metaplasia indicates that the initiation of intestinal metaplasia constitutes a bona fide genetic bottleneck event. Henceforth, all genetic changes present within the emerging metaplastic stem cell lineage are carried forward as the metaplastic patch expands within the chronically inflamed mucosa. Crucially, the accumulation of random genetic changes within gastric epithelial stem cells leads to each metaplastic patch being endowed with a completely unique repertoire of genetic changes. These mutations may further support either the clonal expansion (proliferation) or the persistence (survival) of metaplastic clones. A follow-up study confirmed the clonal origin of intestinal metaplasia and revealed that areas of dysplasia were genetically related (sharing the same mutations in the *APC* gene) and originated from a single metaplastic clone ^20^. The above scenario describes the clonal selection and expansion of a single focus of intestinal metaplasia ( Figure 2). This scenario is played out many times in the chronically inflamed stomach, signalling the erratic emergence of numerous clones and subclones, dividing the gastric mucosa into a mosaic of competing clones battling for space in a crowded niche. All of these individual patches may expand through gland duplication (also known as gland fission) ^19^ and coalesce to cover large swathes of the gastric mucosa ( Figure 1).

Histologically, GIM can be subdivided into either complete or incomplete intestinal metaplasia. Complete IM resembles small intestinal glands, with loss of gastric mucins (MUC1, MUC5AC, and MUC6) and the presence of eosinophilic enterocytes with an identifiable brush border, well-defined goblet cells, and occasional Paneth cells at the base of the gland. Incomplete intestinal metaplasia (also known as gastric or mixed-type intestinal metaplasia) shows a combination of gastric foveolar epithelium and intestinal goblet cells with simultaneous expression of both gastric and intestinal mucins ^21^. It has been demonstrated that the incomplete form of intestinal metaplasia has a higher proliferative index ^22^. The prognostic implications, if any, of these subtypes of intestinal metaplasia remain uncertain; however, retrospective longitudinal studies have suggested a higher risk of progression to cancer with the incomplete type ^23, 24^, indicating that it may either represent a more advanced form of intestinal metaplasia or be the result of a separate evolutionary pathway. Further work, in particular further research on the possible clonal ancestry of complete and incomplete intestinal metaplasia, is required to substantiate these claims. A second form of metaplasia, spasmolytic polypeptide-expressing metaplasia (SPEM), also known as pseudo-pyloric metaplasia, has been implicated as a condition with an increased risk of progression to intestinal-type gastric cancer ^25– 27^. SPEM is morphologically similar to antral glands with loss of parietal cells and expression of MUC6 and TFF2. A greater than 90% prevalence of SPEM in the background mucosa of resected gastric cancer has been seen ^27^, with recent work suggesting advanced SPEM had a stronger association with both EBV-positive and -negative gastric cancer ^28^. This work suggests that the identification of SPEM may be considered a risk indicator for cancer development at least similar to the observation of intestinal metaplasia. Again, whether SPEM can progress to intestinal metaplasia or to gastric cancer without intestinalised precursor is unclear. Clonal lineage tracing on patient material will undoubtedly resolve these issues.

This process of clonal evolution of intestinal metaplasia occurs against a background of progressive glandular atrophy. Atrophy here is described as physical loss of native glandular units and is a common denominator in all pathological processes causing progressive mucosal damage, including longstanding *H. pylori* infection ^29^. Atrophy occurs either as complete loss of native glands with resulting fibrosis (direct atrophy) or as the replacement of native glands with either pseudo-pyloric metaplasia or intestinal metaplasia (functional atrophy). *H. pylori* infection is the major aetiological driver for atrophy, although corpus-predominant atrophy can be seen in a subset of autoimmune gastritides ^30^. In a study by El-Zimaity *et al.*, the progression of gastric atrophy was mapped in detail across resection specimens. The authors showed that the progression of gastric atrophy occurs in the form of pseudo-pyloric metaplasia (also known as SPEM) as a continuous sheet, with or without intervening islands of intestinal metaplasia ^31^. These analyses suggest that gastric atrophy spreads in the form of pseudo-pyloric metaplasia from the pylorus proximally like a moving wave-front, along the lesser curve, and into the anterior and posterior walls of the stomach. This metaplastic replacement may be accompanied by the physical loss of glands with fibrosis (direct atrophy). Further work has indicated that there is increased *H. pylori* colonisation and active inflammation just proximal to this atrophic wave-front, compared to mucosa immediately distal to the wave-front. Furthermore, the mucosa directly distal to the wave-front shows atrophy and intestinal metaplasia as a form of mucosal ‘scarring’ ^32^. *H. pylori* thrives only within a narrow pH range, and this selectivity, in combination with host factors ^33^, may be responsible for this dynamic ‘moving wave-front’ pattern of expansion from distal to proximal along the gastric mucosal surface ^34^.

The role of *H. pylori* infection in this sequence is to drive a catastrophic change in the gastric mucosal ecology. Indeed, *H. pylori* is generally absent from areas with intestinal metaplasia, and this correlates with decreased mucosal inflammation scores ^35^. These data suggest that the change in epithelial phenotype from gastric to intestinal columnar carries a distinct selective advantage, which drives the progressive expansion of patches of intestinal metaplasia. In this way, increased mutation rate and natural selection, the two core driving forces of Darwinian evolution, collude to drive progression to cancer in the chronically inflamed stomach.

Further genetic diversification inevitably occurs whilst patches of intestinal metaplasia clonally expand and compete, until transformation to dysplasia and ultimately gastric adenocarcinoma occurs. Recognising the mosaic development of gastric adenocarcinoma, in the context of CAG, as a branching evolutionary process rather than a linear cascade allows us to understand the dynamic progression to cancer in the chronically inflamed stomach in new and powerful ways. Parenthetically, whether or not intestinal metaplasia is an obligate precursor to gastric cancer, as stipulated by the Correa sequence, remains to be definitively shown. We have recently shown in the metaplastic distal oesophagus that oesophageal adenocarcinoma can clonally derive from pre-malignant clonal expansions in non-intestinalised columnar epithelium ^36^. This at least suggests that gastric cancer may in a similar fashion derive directly from pseudo-pyloric metaplasia without intestinalised intermediate. This would suggest that intestinal metaplasia is definitely a risk marker and a potential precursor but not the sole or obligate precursor to gastric cancer. This further highlights the importance of a branching evolutionary model for gastric cancer progression. Mapping these dynamic topographic patterns through enhanced imaging modalities and targeted histopathology may therefore facilitate more accurate risk stratification of the chronically inflamed stomach.

## Risk factors for the development of chronic atrophic gastritis and gastric intestinal metaplasia and progression to cancer

Prior to performing an upper GI endoscopy, there are several risk factors that should be considered in order to gauge a patient’s potential risk of having CAG and GIM. The link between *H. pylori* and gastric cancer is well recognised ^12^, with both CAG and GIM being a common finding in those with previous *H. pylori* infection, but serological studies suggest that the association with CAG may be underestimated because of clearance of the infection in advanced stages of CAG ^37^ and the known inter-observer variability for histopathologic scoring of CAG.

*H. pylori* infection provokes chronic inflammation and the production of reactive oxygen and nitrogen species, leading to oxidative genetic damage ^38^. Environmental, host, and bacterial factors all contribute to an individual’s likelihood of *H. pylori* infection progressing to ** atrophic gastritis and subsequent gastric cancer. A number of bacterial virulence factors have been identified, with those expressing genes of the cag (cytotoxic-associated antigen) pathogenicity island, e.g. the *cagA* gene, triggering an enhanced inflammatory response and therefore an increased likelihood of significant outcomes ^38^. Polymorphisms in host genes that regulate the inflammatory response to *H. pylori* infection, e.g. interleukins (IL1B, IL1RN, IL8, and IL10), can result in a pro-inflammatory genotype and may explain regional and ethnic variations in cancer prevalence. This has been extensively reviewed elsewhere ^39^.

There is an increased incidence of CAG in those with a family history of gastric cancer ^40^. Furthermore, a meta-analysis described an odds ratio (OR) of 1.982 for the presence of GIM in first-degree relatives of gastric cancer patients ^41^. Pernicious anaemia is associated with a higher risk of CAG and GIM, and a recent meta-analysis estimated the pooled risk of developing gastric cancer at 0.27% per person-years ^42^. Several studies have demonstrated increased risk of CAG and GIM in male smokers ^43– 46^ and those with a high-salt diet ^47^. Advancing age remains a key risk factor for the development of CAG, GIM, and subsequent gastric adenocarcinoma, with three studies showing that patients over 45 years old have an OR of between 1.92 and 3.1 for the progression of the premalignant stomach to cancer ^43, 48, 49^. Longitudinal population studies have examined the risk of cancer associated with CAG and GIM. A Dutch study included all patients with histopathological diagnoses of CAG and/or GIM and described annual incidences of gastric cancer of 0.1% and 0.25%, respectively ^48^. More recently, Song *et al.* examined a retrospective cohort of 3,714 patients with confirmed CAG and demonstrated that the extent of atrophy and intestinal metaplasia, age over 55 years, and alcohol consumption were significant risk factors for the development of gastric neoplasia ^50^. It is also clear that ethnicity and geographic location are determinants of gastric cancer risk in the setting of CAG and GIM. A systematic review by Spence *et al.* revealed a higher gastric cancer incidence in those diagnosed with CAG and/or GIM in East Asian countries ^51^, while a recent US study showed an ongoing elevated risk of gastric cancer in immigrant populations of East Asian origin living in the USA ^52^. Finally, Spence *et al.*, in a meta-analysis, also reported significant variation of gastric cancer incidence with CAG and GIM but additionally noted that, in general, study quality was poor and there was marked heterogeneity between studies (I ^2^ statistic of ±95%) ^51^.

**Figure 3.**
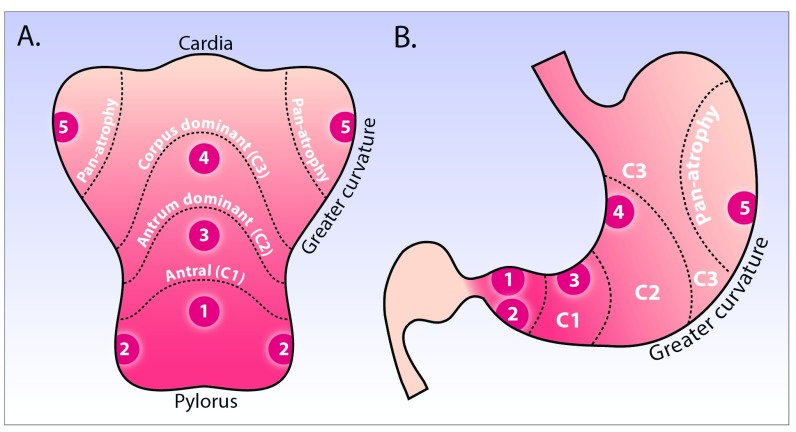
Updated Sydney and modified Kimura-Takemoto classification systems for histopathologic and endoscopic staging of chronic atrophic gastritis. **A**) and **B**) show the stomach opened along the greater curvature ( **A**) and in traditional coronal view ( **B**). Depicted is endoscopic atrophy grading according to the modified Kimura-Takemoto classification system ^60^: antral (C-1), antral-predominant (C-2), corpus-predominant (C-3), and pan-atrophy. The circled numbers 1–5 correspond to the location of gastric biopsies, which should be taken according to the updated Sydney system: antrum greater and lesser curve, incisura, and corpus greater and lesser curve.

The grade and severity of gastric atrophy is predictive of gastric cancer risk. Japanese data showed that when compared to subjects with no or mild atrophy, patients with moderate atrophy carried a relative risk of gastric cancer at 1.7, but this rose to a relative risk of 4.9 in those with severe gastric mucosal atrophy ^53^. The OLGA (operative link for gastritis assessment) and OLGIM (operative link on GIM) systems have been advocated for the staging of gastritis ^54, 55^. Individuals with stage III or IV CAG or GIM, based on these systems, are at increased cancer risk ^54^. At present, European Society of Gastrointestinal Endoscopy management of precancerous conditions and lesions in the stomach (ESGE MAPS) guidelines ^56^ recommend 3-yearly surveillance for those with extensive CAG or GIM, with random biopsies taken as per the geographic locations of the updated Sydney protocol, and histological samples staged using the OLGA/OLGIM systems ( Figure 3A and B). This approach has inherent limitations; in clinical practice, these systems can be difficult to apply, the OLGA system is limited by considerable inter- and intra-observer variability, and, while the OLGIM system reduces this with increased inter-observer agreement ^55^, it may nonetheless lead to over- or under-diagnosis due to random biopsy sampling error ^54, 57, 58^. In short, while higher stages of CAG and GIM are predictive of increased gastric cancer risk, current histopathologic staging methods to risk stratify CAG patients are all fraught with significant drawbacks, which likely explains widely varying estimates between studies.

By contrast, long-term cohort studies suggest that the endoscopic Kimura-Takemoto classification scheme has significant utility as a risk stratification tool to predict gastric adenocarcinoma risk ^53, 59^. This system classifies gastric atrophy into six endoscopic stages according to the location of the endoscopic atrophic border. It requires considerable endoscopic experience to be used effectively and has not been widely adopted by Western endoscopists. A modified Kimura-Takemoto classification ( Figure 3A and B) has been proposed ^60^, with gastric atrophy simplified to three grades: normal (no atrophy), limited (antral and antral-predominant atrophy), and extended (corpus-predominant and panatrophy). With this system, endoscopic and histological scores of atrophy showed complete concordance in 69.8%, and the strength of agreement of the extent of atrophy between histology and endoscopy showed good reproducibility with a weighted kappa value of 0.76 (95% CI 0.71–0.80) ^60^. Our working group is currently investigating the accuracy and reproducibility of a simplified endoscopic scoring system, for use in Western practice, in an endoscopy-led staging paradigm for chronic gastritis.

## Optimal techniques for the endoscopic detection and classification of chronic atrophic gastritis and gastric intestinal metaplasia

The first step to being confident in the diagnosis and assessment of gastric premalignant changes, and indeed early cancer, is high-quality endoscopy. Although there is limited evidence in this field, the ESGE have outlined a number of principles in their recent statement on upper GI endoscopy (ESGE 2016 upper GI performance measures) ^61^. The key performance measure of a minimum 7-minute procedure time, for first diagnostic upper GI endoscopy and follow up of GIM, is based on retrospective data ^62^ showing that longer procedures (>7 minutes) were twice as likely to detect high-risk gastric lesions, defined as biopsy-proven GIM, CAG, gastric dysplasia, or cancer (OR 2.50, 95% CI 1.52–4.12), and three times as likely to detect dysplasia or cancer (OR 3.42, 95% CI 1.25–10.38).

### Endoscopic features of chronic atrophic gastritis and gastric intestinal metaplasia using white light endoscopy

Macroscopically, as the atrophy in CAG progresses, the gastric folds disappear. This loss of gastric rugae combined with mucosal pallor and increased visibility of mucosal vessels constitute the main endoscopic features of atrophic gastritis ^63, 64^. Increased visibility of the vascular network showed a sensitivity of 48% and a specificity of 87%, while the loss of gastric folds has a sensitivity of 67% and a specificity of 85% ^65^.

**Figure 4.**
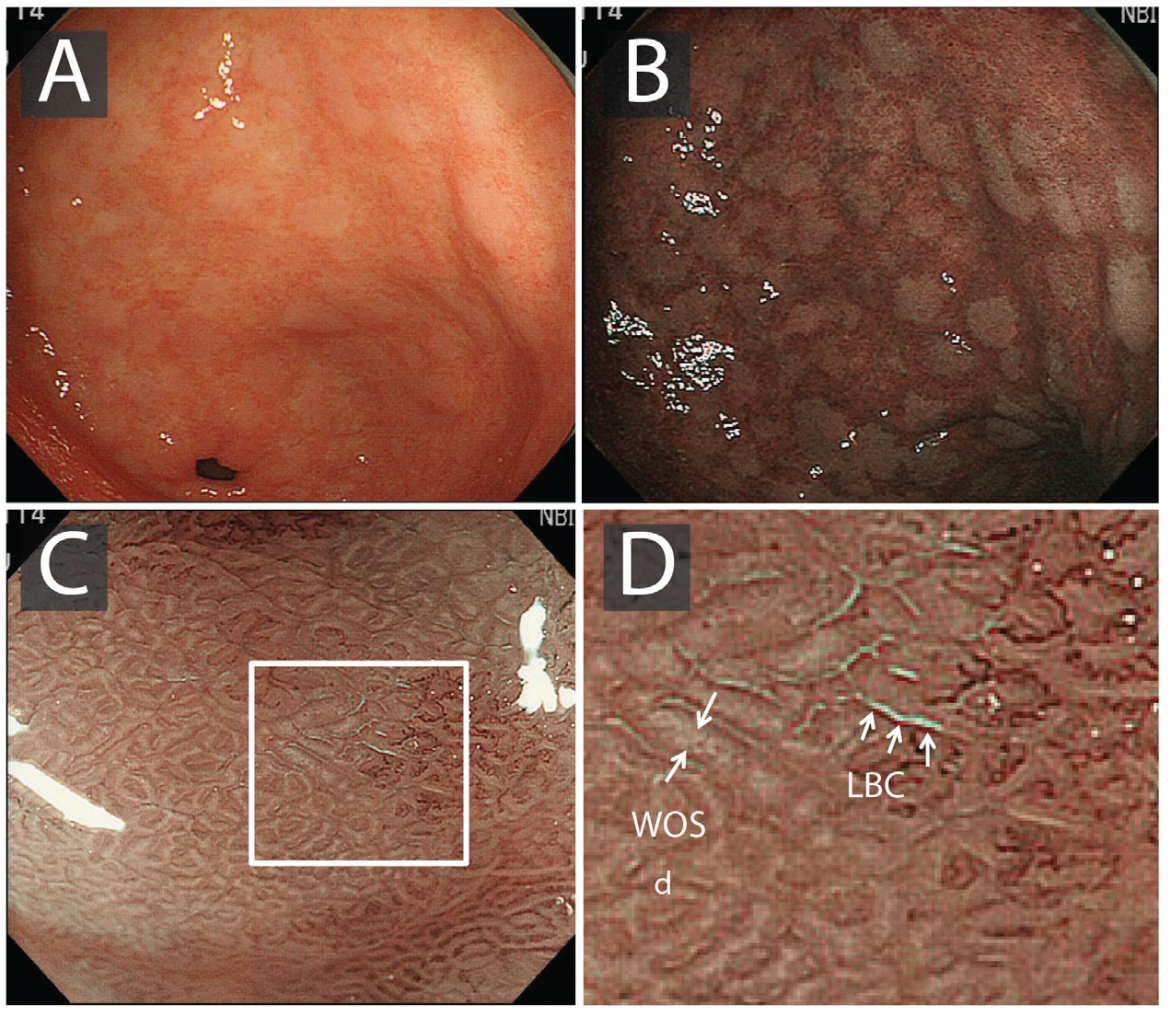
Endoscopic findings of gastric intestinal metaplasia. **A**) White light endoscopy image, with gastric intestinal metaplasia clearly visible as pale whitish patches. **B**) Narrow band imaging enhances mucosal contrast, highlighting patches of gastric intestinal metaplasia. **C**) Magnification endoscopy demonstrates light-blue crests (LBCs) on the surface of the epithelium; in some cases, a white opaque substance (WOS) is seen in the intervening part of the crypt openings. **D**) Corresponds with the white square in image **C**. Images from 63.

At white light endoscopy (WLE), intestinal metaplasia typically appears as small, grey-white, elevated plaques surrounded by mixed patchy pink and pale areas of mucosa causing an irregular uneven surface ( Figure 4). Mottled patchy erythema has also been positively associated with intestinal metaplasia ^66^. The diagnosis of GIM using standard WLE alone is of inferior accuracy to narrow band imaging (NBI). Strikingly, Pimental-Nunes *et al*. showed significant improvement in accuracy with NBI versus WLE for the detection of intestinal metaplasia (87% versus 53%; *p*< 0.001) ^67^, and other studies have suggested that WLE is of insufficient reliability to be an acceptable sole diagnostic modality for GIM ^67^.

### Enhanced endoscopy: magnification and virtual chromoendoscopy

High-resolution magnification endoscopy has been shown to reliably identify normal gastric mucosa, *H. pylori-*associated gastritis, and gastric atrophy ^68^. As patches of intestinal metaplasia expand, the glands elongate to form a ‘groove-type pattern’ similar to that of the antrum or villiform pattern of the intestine. Although these changes can easily be distinguished from the normal mucosa in the corpus, intestinal metaplasia in the antrum is more difficult to characterise ^63, 69^. Additional features of GIM include the light-blue crest (LBC) and the marginal turbid band (MTB) ^70, 71^. Using NBI with magnifying endoscopy (NBI-ME), the LBC appears as a fine, blue-white line on the crest of the epithelial surface and is a highly accurate sign for the presence of intestinal metaplasia at histology ^69– 71^ ( Figure 4). A white opaque substance (lipid droplets) obscuring the subepithelial capillaries is another endoscopic finding associated with intestinal metaplasia ^64^.

Comparison of NBI with WLE has shown superior detection rates for CAG and GIM ^72, 73^. A recent meta-analysis found that NBI-ME had a very high diagnostic efficacy for diagnosing early gastric adenocarcinoma (pooled sensitivity 0.83 [95% CI 0.79–0.87, I ^2^=79.8%] and pooled specificity 0.96 [95% CI 0.95–0.97, I ^2^=89.3%]) ^74^. Buxbaum *et al*. compared the detection of GIM from biopsies directed by NBI with those directed by high-definition WLE (HD-WLE) and those taken from mapping biopsies. This was performed in a blinded fashion by different endoscopists. Their results suggested the highest yield of GIM comes from a combination of NBI-directed biopsies and mapping biopsies ^75^. A caveat to this conclusion was that the staging was performed during the same procedure. A simplified classification system using NBI without magnification by Pimentel-Nunes *et al.* has proved to be accurate and reliable for the diagnosis of intestinal metaplasia and dysplasia. In this validation study, a tubulo-villous mucosal pattern was associated with intestinal metaplasia (accuracy 84%; 95% CI 77–91%; positive likelihood ratio LR+=4.75), while irregular vessels and mucosal pattern was associated with dysplasia (accuracy 95%; 95% CI 90–99%; LR+=44.33). The LBC finding was moderately reliable (k=0.49) but specific (87%) for intestinal metaplasia ^76^.

Pimentel-Nunes *et al.* showed that NBI demonstrated a high concordance with histopathological diagnosis, superior to standard WLE ^67^. Diagnostic accuracy higher than 90% suggests that routine use of NBI allows targeted instead of random biopsy samples. However, it is important to note that this study assessed WLE plus NBI, rather than NBI alone, and although HR WLE had a good overall sensitivity of 85% for all pathology, this decreased to 53% for the detection of GIM. Xirouchakis *et al*. reported better WLE accuracies in combination with updated Sydney protocol biopsies for CAG and GIM (93% and 90%, respectively) when compared to NBI (80% and 82%, respectively) ^77^.

A scale for the endoscopic grading of GIM using NBI was created and returned an area under the curve of 0.98 for WLE followed by NBI for diffuse GIM ^67^. It is noteworthy that in this study the accuracy of NBI was assessed by one endoscopist who had previously graded the same gastric mucosa with HD-WLE rather than NBI alone. It cannot be excluded that this unblinded study design may have affected the study’s outcome. It should also be noted that these exceptional results were obtained by highly experienced endoscopists who were all performing >100 NBI procedures per year. Nevertheless, on this basis, it can be argued that endoscopic staging with HD-WLE plus NBI is sufficiently accurate for the endoscopic diagnosis and staging of GIM, provided the assessment is carried out by an appropriately experienced endoscopist.

A recent systematic review by Kikuste *et al.* found that a ridge/tubulo-villous mucosal pattern was associated with GIM with a pooled sensitivity, specificity, and diagnostic OR of 0.86 (95% CI 0.82–0.90), 0.77 (95% CI 0.73–0.80), and 17.01 (95% CI 1.4–207.2), respectively ^78^. Both NBI and FICE (Fuji Intelligent Chromo Endoscopy) showed promising results at the characterisation level; however, this is limited by minimal data addressing inter-observer agreement or reproducibility. Most of the studies (90%) were performed using NBI with magnification for intestinal metaplasia in six studies (75%) and for dysplasia in 10 studies (83%). However, with these endoscopes rarely available in Western centres, it remains difficult to generalise these results. A study by Shi *et al.* combined autofluorescence imaging (AFI) with NBI. The sensitivity and specificity of AFI-NBI combined were 88.89% and 91.58%, respectively, in the diagnosis of intestinal metaplasia, 83.33% and 98.51%, respectively, for dysplasia, and 90.91% and 99.22%, respectively, for early gastric cancer, suggesting that, if more widely available in the future, this technique may have a role in complementing our armoury of diagnostic modalities ^79^.

### Novel and emerging endoscopic technologies

The limitations with WLE in detecting and staging the often-subtle patchy field changes seen in GIM and the reliance on histopathology for the confirmation of diagnosis make this a target of interest for new and emerging endoscopic technologies. Confocal laser endomicroscopy (CLE) is not yet routinely used or widely available in Western centres. CLE is an imaging tool allowing the visualisation of the mucosa at cellular resolution (x1,000) in real time. This raises the future possibility of bypassing the need for histopathological confirmation. Its utility in detecting GIM and CAG was examined in two meta-analyses of four and 23 studies, all performed in East Asia ^80, 81^, with pooled sensitivity, specificity, and area under the curve of 92% (90–94%), 97% (96–98%), and 0.9774, respectively, although both sensitivity ( *p*=0.0141, I ^2^=56.5%) and specificity ( *p*=0.0000, I ^2^=90.6%) were heterogeneous between studies. Linked colour imaging (LCI) and blue laser imaging (BLI) are novel image-enhanced endoscopy technologies developed by Fujifilm Corporation (Tokyo, Japan). A study by Kanzaki *et al*. demonstrated enhanced colour differences with LCI between early gastric cancer and surrounding mucosa, especially in the setting of background GIM ^82^. This suggests that LCI may have a role in improving the detection of early cancer in the setting of GIM; further studies are needed to validate this tool. Full spectrum endoscopy (FUSE; EndoChoice, Inc., Alpharetta, GA, United States) provides a 245-degree field of view with double imagers on the front and side of the endoscope tip. Data are emerging in the colon, and it has been shown to be usable and feasible in a pilot study for upper endoscopy ^83^, but its role in detecting gastric pathology remains undefined. Deep-learning algorithms with construction of convolutional neural networks to classify endoscopic images is an emerging field, with early data suggesting efficacy for distinguishing imaging data for Barrett’s oesophagus and Barrett’s-related neoplasia ^84^. It remains to be seen how this will be adapted to fit the complex mosaic field of changes of the chronically inflamed stomach.

### Biopsy strategies

The latest Kyoto Consensus report in 2015 ^85^ outlines the global consensus on the diagnosis and risk stratification of chronic gastritis. Biopsies should always be undertaken in patients with an endoscopic suspicion of CAG, GIM, or early neoplasia. Using WLE, features such as the absence of collecting venules, loss of gastric folds, presence of an atrophic border, and increased visibility of mucosal vessels should prompt biopsies ^86^. A groove-type and villiform pattern can usually be seen on high-resolution WLE with the addition of NBI, iScan (Pentax, Image Enhanced Endoscopy), or FICE but more easily seen with the addition of magnification. Magnifying NBI can further highlight the LBC sign ^70, 71^. If any of these features are present, biopsies should be taken for histopathologic confirmation.

**Figure 5.**
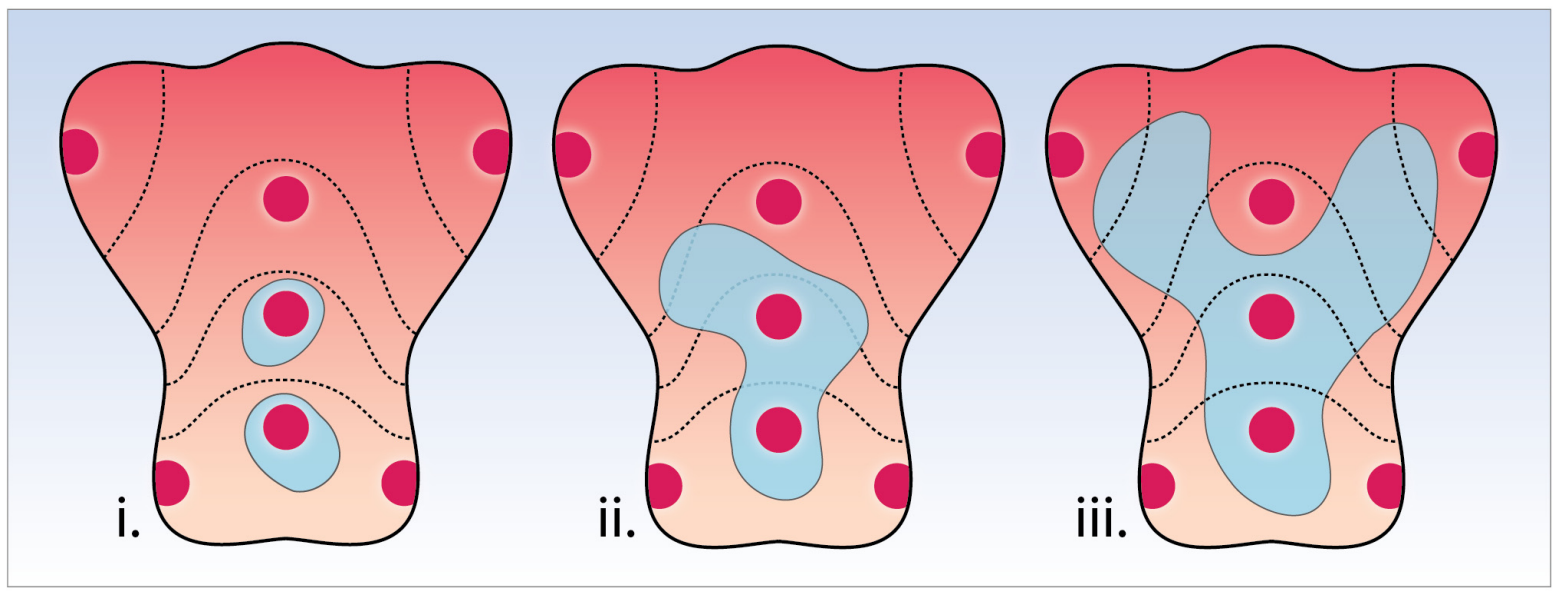
Random biopsy sampling according to the updated Sydney protocol carries the risk of sampling error. In this hypothetical example, three patients are shown, each of whom would be reported as consistent with limited atrophy, as only two of the random Sydney biopsy locations are positive for intestinal metaplasia. However, the geographical spread of intestinal metaplasia (in grey) varies widely between these patients, clearly illustrating an important drawback of random biopsy protocols.

Current surveillance protocols stipulate random biopsies in areas according to the updated Sydney protocol. However, random sampling does not reliably foster the correlation of endoscopic and histopathologic findings and carries significant risk of sampling error, leading to inaccurate or missed diagnosis ( Figure 5). Our understanding that the chronically inflamed stomach progresses through a mosaic of expanding patches of intestinal metaplasia that eventually coalesce reinforces the complexity of the pathogenesis to cancer and the drawbacks of a random biopsy-led protocol. By contrast, studies reviewed here are now suggesting that the endoscopic detection and grading of GIM is accurate ^67^ and the aforementioned numeric classification for the staging of GIM has been shown to correlate strongly with OLGIM and with the extent of GIM ^76^, supporting a more disease-tailored, directed (targeted) biopsy strategy.

Endoscopists should therefore be encouraged to 1. make an endoscopic assessment of the presence and extent of GIM, 2. document these findings using the (simplified) Kimura-Takemoto system, and, finally, 3. obtain targeted biopsies from foci endoscopically suspicious for GIM in areas of the updated Sydney protocol. This will drive quality improvement through improved accuracy and reliability of staging, allowing the endoscopist to guide the histopathologist in patient risk stratification and earlier detection of premalignant gastric lesions.

## Conclusion

Recognition and accurate, robust assessment of the chronically inflamed stomach is essential to the early diagnosis of gastric cancer. In an era where significant advances have been made in endoscopic imaging, emerging data suggest that, by embracing widely available tools such as HD endoscopy and image enhancement (NBI), a dedicated endoscopist is equipped with the ability to diagnose and stage premalignant lesions in the stomach (CAG and GIM) with sufficient accuracy to support an endoscopy-led staging system. Targeted biopsies will complement this approach by confirming endoscopic staging. An endoscopic staging paradigm for the premalignant stomach will drive quality improvement and allow patients to benefit from improved diagnostic accuracy, leading to the development of an evidence-based surveillance strategy for the high-risk stomach. We believe that this will drive further research and enable earlier recognition and treatment of gastric cancer, with the aim of reducing the overall cancer-related mortality.

## Abbreviations

AFI, autofluorescence imaging; CAG, chronic atrophic gastritis; CLE, confocal laser endomicroscopy; EBV, Epstein-Barr virus; FICE, Fuji Intelligent Chromo Endoscopy; GI, gastrointestinal; GIM, gastric intestinal metaplasia; HD, high definition; LBC, light blue crest; LCI, linked colour imaging; NBI, narrow band imaging; NBI-ME, narrow band imaging with magnifying endoscopy; OLGA, operative link for gastritis assessment; OLGIM, operative link for gastric intestinal metaplasia; OR, odds ratio; SPEM, spasmolytic polypeptide-expressing metaplasia; WLE, white light endoscopy.
